# Starfish-Derived Extracts Enhance Mitophagy and Suppress Senescence-Associated Markers in Human Dermal Fibroblasts

**DOI:** 10.3390/md23110418

**Published:** 2025-10-27

**Authors:** Hyun Jung Lee, Junhee Kim, Bada Won, Dong Hun Lee, Ok Sarah Shin

**Affiliations:** 1 BK21 Graduate Program, Department of Biomedical Sciences, College of Medicine, Korea University Guro Hospital, Seoul 08308, Republic of Korea; 2 R&D Center, Star’s Tech Co., Ltd., 28, Digital-ro 30-Gil, Guro-gu, Seoul 08389, Republic of Korea; 3 Department of Dermatology, Seoul National University Hospital, Seoul National University College of Medicine, Seoul 03080, Republic of Korea; 4 Institute of Human-Environment Interface Biology, Medical Research Center, Seoul National University, Seoul 03080, Republic of Korea

**Keywords:** starfish-derived extracts, mitophagy, senescence, skin aging, human dermal fibroblasts

## Abstract

While the starfish species *Asterias pectinifera* (*Ap*) and *Asterias amurensis* (*Aa*) are considered ecological threats to marine environments and the fishing industry, recent studies have identified them as rich sources of highly water-soluble, non-toxic collagen peptides. Mitochondrial dysfunction is a key driver of cellular senescence and skin aging, yet the therapeutic potential of marine-derived extracts in modulating mitophagy remains largely unexplored. In this study, we investigated whether starfish-derived extracts could mitigate senescence-associated phenotypes in human dermal fibroblasts (HDFs) through the modulation of mitophagy. Treatment with *Ap*- or *Aa*-derived extracts led to reduced senescence-associated β-galactosidase (SA-β-gal) activity, decreased expression of matrix metalloproteinase-1 (MMP-1), and suppression of pro-inflammatory cytokines including interleukin-6 (IL-6) and interleukin-8 (IL-8). *Ap*- or *Aa*-derived extracts significantly increased mitophagy in HDFs stably expressing mitochondrial-targeted Keima (HDF-mtKeima), while knockdown of PINK1, the essential regulator of mitophagy, abolished the mitophagy-inducing effects of *Ap*- or *Aa*-treatment, indicating that *Ap*- or *Aa*-derived extracts activate PINK1/Parkin-dependent mitophagy pathways. Importantly, PINK1 knockdown reversed starfish-induced suppression of MMP-1 and p21, demonstrating its crucial role in regulating senescence-associated gene expression. Additionally, *Ap* or *Aa* treatments significantly reduced reactive oxygen species (ROS) accumulation, improved mitochondrial function, and enhanced both basal and maximal respiratory capacity in senescent HDFs. These findings highlight that extracts derived from starfish promote mitophagy through PINK1-dependent mechanisms, exhibiting significant anti-senescence effects in HDFs. This suggests their potential application in the development of novel cosmeceuticals with skin-protective and rejuvenating properties.

## 1. Introduction

From intertidal to deep-sea settings, starfish can be found in all of the world’s oceans. Starfish is considered a harmful species as it is capable of devouring a variety of marine life, including fish and seaweed, and thus is thought to pose a serious threat to the health of marine ecosystems as well as the livelihoods of the fishing and aquaculture sectors. However, starfish-derived extracts can be a source of different low-molecular-weight chemicals, providing the potential to offer anti-aging, anti-inflammatory, anti-cancer, and antioxidant qualities [1,2,3,4,5]. We recently reported that the conjugation of starfish-derived collagens with a liposome drug delivery system showed that *Asterias pectinifera* (*Ap*)-derived collagen peptide-encapsulating nanoliposomes showed a high skin absorption rate [6,7]. However, it is unknown whether starfish-derived extracts have the potential to modulate cellular senescence and skin aging.

Skin aging is a complex, multifactorial process regulated at both the epidermal and dermal levels [8]. Dermis aging involves extracellular matrix degradation, decreased collagen synthesis, and fibroblast senescence. A central contributor to this process is cellular senescence, characterized by irreversible cell cycle arrest, altered metabolic activity, and the secretion of pro-inflammatory senescence-associated secretory phenotypes (SASPs) [9]. One of the key drivers of senescence is defective mitochondria, which leads to increased reactive oxygen species (ROS), impaired ATP production, and activation of potent inflammatory reactions [10]. Mitophagy contributes to mitochondrial quality control mechanisms by eliminating dysfunctional mitochondria via autophagy and serves to prevent uncontrollable inflammation [10,11]. PTEN-induced kinase 1 (PINK1) is a serine/threonine kinase that plays a pivotal role in maintaining mitochondrial integrity and cellular homeostasis. Under physiological conditions, PINK1 is imported into healthy mitochondria and rapidly degraded. However, upon mitochondrial membrane depolarization, PINK1 accumulates on the outer mitochondrial membrane, where it initiates a signaling cascade that promotes the selective removal of dysfunctional mitochondria via mitophagy. PINK1/Parkin-mediated mitophagy helps suppress inflammation by preventing the accumulation of dysfunctional mitochondria that would otherwise activate immune sensors [12,13,14]. Mitophagy plays a pivotal role in regulating skin cell senescence, especially under stress conditions like UV exposure [15,16,17,18,19,20]. When mitophagy is impaired, damaged mitochondria accumulate, leading to increased oxidative stress and the promotion of cellular senescence, a hallmark of aging skin.

In this study, we demonstrate that extracts derived from *Asterias pectinifera* (*Ap*) and *Asterias amurensis* (*Aa*) reduce senescence-associated phenotypes in a senescent human dermal fibroblast (HDF) model. This effect is attributed to the suppression of senescence-associated β-galactosidase (SA-β-gal) activity, matrix metalloproteinase-1 (MMP-1), and pro-inflammatory cytokines, including interleukin-6 (IL-6) and interleukin-8 (IL-8). Furthermore, we evaluated the impact of *Ap* or *Aa*-derived extracts on mitophagy in HDFs and found that these marine-derived compounds significantly promote mitophagic activity. These findings suggest that starfish-derived extracts exert anti-aging effects through the enhancement of mitophagy and the attenuation of cellular senescence, highlighting their potential application in the development of cosmetic products with skin-protective and rejuvenating properties.

## 2. Results

### 2.1. Asterias pectinifera (Ap) and Asterias amurensis (Aa) Treatment Reduce Senescence in Senescent Human Dermal Fibroblasts

**Figure 1:**
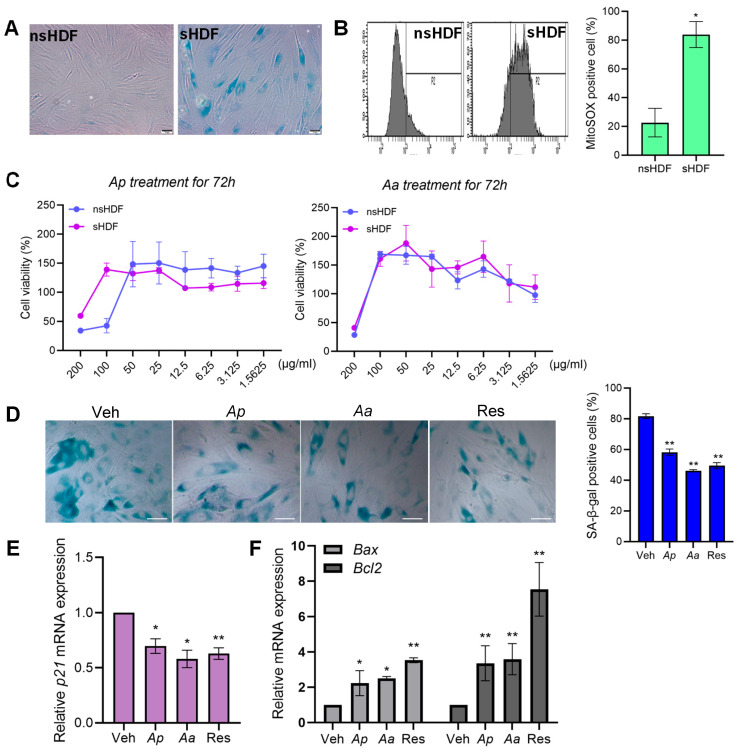
The effect of starfish-derived extracts on cellular senescence of human dermal fibroblasts (HDFs). (**A**) The senescence-associated (SA) β-galactosidase activity of non-senescent (ns) nsHDF (2–7 passages), and senescent (s) HDF (>20 passages). Scale bar = 10 μm (**B**) Mitochondrial ROS (mtROS) was measured by MitoSOX Green staining and mtROS levels were quantified using flow cytometry. * p < 0.05 vs. nsHDF. (**C**) Cells were treated with various concentrations of *Asterias pectinifera* (*Ap*) and *Asterias amurensis* (*Aa*)-derived extracts for 72 h. The cell viability was quantified by cell counting kit-8 (CCK8) assay. (**D**) sHDF were treated with vehicle (Veh), *Ap*, *Aa* or resveratrol (Res) for 72 h and SA-β-gal staining was performed. Scale bar = 20 μm. (**E**,**F**) *p21*, *Bax* and *Bcl2* expression levels in *Ap*- or *Aa*-treated sHDF were measured by qRT-PCR. Relative expression levels were normalized against GAPDH. All data were indicated as mean ± SD of at least three independent experiments. Statistical analysis: * *p* < 0.05, ** *p* < 0.01 vs. vehicle-treated cells.

To evaluate the anti-aging effects of extracts derived from *Asterias pectinifera* (*Ap*) and *Asterias amurensis* (*Aa*) in human dermal fibroblasts (HDFs), we first established both non-senescent (nsHDF) and senescent (sHDF) cell models. Replicative senescence was induced through serial passaging, with senescence confirmed by SA-β-gal staining and mitochondrial-specific oxidative stress assessed via MitoSOX staining. Proliferative capacity was measured across passages 2–7 for nsHDF and beyond passage 20 for sHDF. As expected, sHDF exhibited a marked increase in SA-β-gal activity and significantly elevated mitochondrial reactive oxygen species (mtROS) levels compared to nsHDF (Figure 1A,B), validating the successful induction of cellular senescence.

Prior to assessing the anti-senescence effects of *Ap* and *Aa*, we evaluated their cytotoxicity in HDFs. Cells were treated with varying concentrations of *Ap* and *Aa* for 72 h, and viability was measured using the CCK-8 assay. While high concentrations (>100 μg/mL) reduced cell viability, doses below 50 μg/mL were non-cytotoxic in both nsHDF and sHDF (Figure 1C).

We then investigated whether *Ap* and *Aa* could regulate the senescence phenotypes in sHDF. Cells were treated with vehicle (Veh), *Ap*, *Aa*, or resveratrol—a polyphenolic antioxidant known for its anti-senescence properties and used here as a positive control [21,22]. Treatment with *Ap*, *Aa* or resveratrol resulted in a significant reduction in the proportion of SA-β-gal-positive cells by more than 20%, along with a noticeable decrease in staining intensity compared to vehicle-treated controls (Figure 1D). Consistent with this, the mRNA expression level of *p21*, a well-known senescence marker, was also reduced following treatment with *Ap* or *Aa* (Figure 1E). In addition, the expression levels of *Bcl-2-associated X protein* (*Bax*) and *B-cell lymphoma 2* (*Bcl-2*), which have opposing roles in apoptosis regulation, were both significantly increased following *Ap* and *Aa* treatment (Figure 1F). Taken together, these findings demonstrate that *Ap* and *Aa*-derived extracts effectively attenuate senescence-associated phenotypes in HDFs.

### 2.2. Anti-Inflammatory Effects of Ap or Aa-Derived Extracts in Human Dermal Fibroblasts

**Figure 2:**
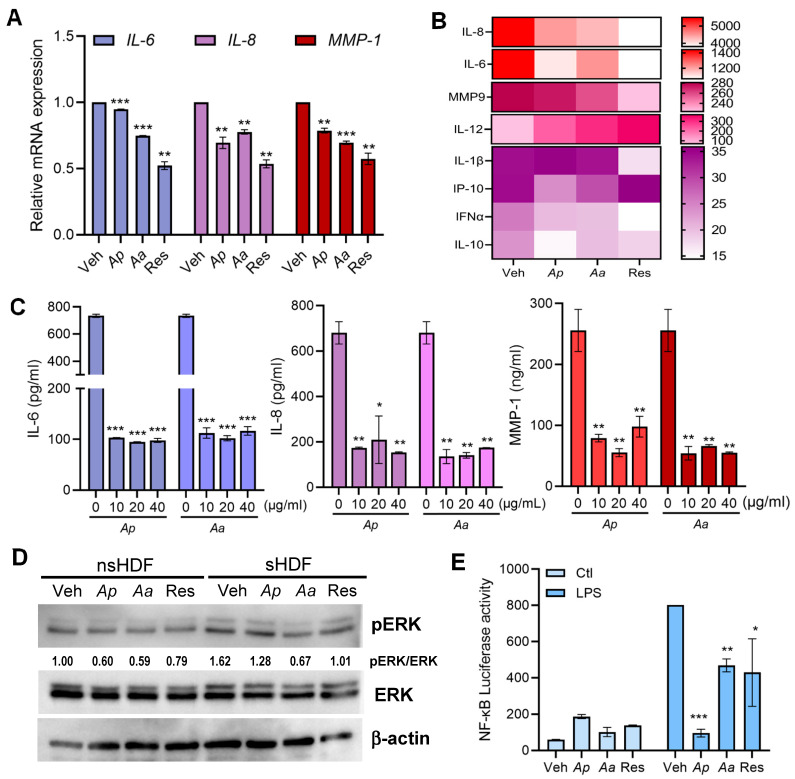
Starfish-derived extracts inhibit inflammatory signaling pathways in HDF. (**A**) sHDF were treated with vehicle (Veh), 10 μg/mL of *Ap*, *Aa* or resveratrol (Res) for 4 h. Relative mRNA expression levels of SASP-related genes *IL-6*, *IL-8* and *MMP-1* were determined by qRT-PCR. Relative expression levels were normalized against GAPDH. (**B**) sHDF were treated with Veh, 10 μg/mL of *Ap*, *Aa* or Res for 24 h and cell supernatants were collected for further analysis. A heatmap shows the expression level of cytokines involved in SASPs by multiplex cytokine assay (Luminex). (**C**) sHDF were treated with 10, 20, or 40 μg/mL of *Ap*, *Aa*, or Veh only for 24 h. Secretion levels of IL-6, IL-8 and MMP-1 were measured by ELISA. (**D**) Cells were treated with Veh, 10 μg/mL of *Ap*, *Aa* or Res for 2 h. Protein levels of phosphorylated and total ERK were analyzed by Western blotting and the numbers below the blot represent relative band intensity. (**E**) HDFs were transfected with NF-κB reporter plasmid for 24 h and cells were treated with Veh, 10 μg/mL of *Ap*, *Aa*, or Res for 24 h followed by 100 ng/mL LPS treatment. A luciferase assay was performed to measure the promoter activity of NF-κB reporter plasmid. All the data are presented as mean ± SD from *n* = 3. Statistical analysis: * *p* < 0.05, ** *p* < 0.01, *** *p* < 0.001 vs. vehicle-treated cells.

To examine whether *Ap* and *Aa* exert anti-inflammatory effects through the modulation of SASP factors in sHDF, we first measured the mRNA expression levels of IL-6, IL-8 and MMP-1 (Figure 2A). Next, we comprehensively analyzed cytokine secretion profiles from sHDF following *Ap-* or *Aa*-derived extracts treatment, using the multiplex cytokine analysis Luminex and ELISA assays (Figure 2B,C). The results showed that *Ap* and *Aa* treatment significantly reduced the transcriptional and secretory levels of IL-6, IL-8, and MMP-1. Next, we aimed to investigate the effects of *Ap* and *Aa* treatment on extracellular signal-regulated kinase (ERK) signaling in HDFs. Following *Ap* and *Aa* treatment, the level of ERK phosphorylation was assessed and quantified using immunoblot analysis (Figure 2D). A decrease in ERK phosphorylation was observed in both nsHDF and sHDF treated with *Ap* or *Aa*. To investigate whether the observed anti-inflammatory effects were mediated through NF-κB–dependent inflammatory signaling, cells were transfected with an NF-κB luciferase reporter plasmid and subsequently treated with lipopolysaccharide (LPS), a known activator of NF-κB [23]. After LPS stimulation, cells were treated with *Ap* or *Aa* to assess whether the starfish extracts could modulate LPS-induced NF-κB transcriptional activity. Figure 2E demonstrates that LPS-induced increase in NF-κB transcriptional activity was markedly attenuated by *Ap* or *Aa* treatment. These findings suggest that *Ap* and *Aa* mitigate SASP-associated inflammation, thereby contributing to their overall anti-senescence activity.

### 2.3. Starfish-Derived Extracts Promote Mitophagy in HDFs

**Figure 3:**
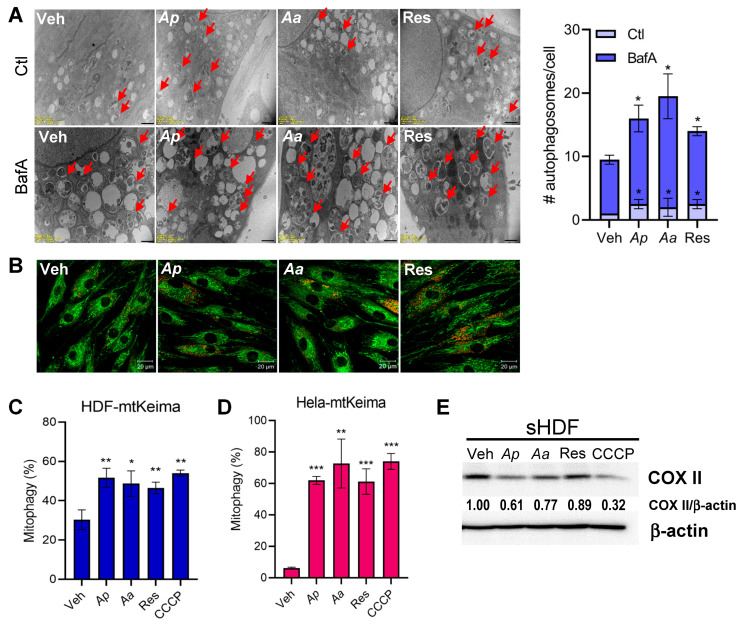
Starfish-derived extracts upregulates mitophagy. (**A**) nsHDF were treated with vehicle (Veh), 10 μg/mL of *Ap*, *Aa*, or resveratrol (Res) for 16 h with or without co-treatment with 100 nM bafilomycin (BafA), and analyzed via transmission electron microscopy. Arrows indicate autophagosomes containing mitochondria. Scale bars = 0.1 μm (bottom). Quantification of autophagosomes per cell is presented on the right as mean ± SD (n ≥ 10 cells per sample). Data represents five independent biological replicates, each expressed as mean ±  SD. (**B**) nsHDF were treated with Veh, 10 μg/mL of *Ap*, *Aa*, or Res for 24 h. Immunofluorescence images showing MitoTracker (green) and LysoTracker (red) staining. Scale bar: 20 μm. (**C**,**D**) HDFs and HeLa stably expressing mitochondria-targeted Keima (HDF-mtKeima, Hela-mtKeima) were treated with *Ap*, *Aa*, or Res for 24 h. Following Ap or Aa treatment, mitophagy induction was subsequently quantified by flow cytometry, based on the red-to-green fluorescence ratio of mtKeima, which reflects mitochondrial delivery to lysosomes. As a positive control of mitophagy induction, cells were treated with 25 μM CCCP for 2 h. (**E**) sHDF cells were treated with Veh, 10 μg/mL of *Ap*, *Aa*, Res, or CCCP for 2 h. Protein levels of cytochrome C oxidase subunit II (COX II) were analyzed by immunoblotting. Anti-β-actin antibody was used as a loading control. All data are presented as mean ± SD from *n* = 3. Statistical analysis: * *p* < 0.05, ** *p* < 0.01, *** *p* < 0.001 vs. vehicle-treated cells.

Recent studies suggest that the accumulation of damaged mitochondria amplifies skin aging phenotypes and the mitophagy, a selective removal process, is crucial for maintaining skin cell homeostasis [24,25]. To investigate the role of starfish-derived extracts on mitophagy, we first quantified whether autophagosomes are formed upon starfish-derived extract treatment in sHDF. To directly visualize autophagosomes, transmission electron microscopy (TEM) was employed. As expected, bafilomycin A1 (BafA) treatment markedly increased the number of autophagosomes, and Figure 3A demonstrates a further accumulation of autophagosomes containing fragmented mitochondria in starfish-treated sHDF, suggesting enhanced autophagic activity. To further assess mitophagy activation, we employed g MitoTracker and LysoTracker co-staining to visualize mitochondrial-lysosomal structures indicative of mitophagic flux. Notably, *Ap-*, *Aa-*, and resveratrol-treated cells exhibited enhanced colocalization of lysosomal (red) puncta with mitochondria, compared to vehicle-treated controls, confirming the induction of mitophagy by starfish-derived extracts (Figure 3B). To further quantify mitophagy, we generated HDF cells stably expressing mitochondria-targeted Keima (mtKeima; HDF-mtKeima) to measure the level of mitophagy induction following *Ap* or *Aa* treatment. As a positive control for mitophagy activation, cells were treated with 25 μM carbonyl cyanide m-chlorophenyl hydrazone (CCCP) for 2 h, a well-established inducer of mitochondrial depolarization and PINK1/Parkin-mediated mitophagy. We confirmed an approximately two-fold increase in the level of mitophagy activation with *Ap*-, *Aa*-, or resveratrol–treated cells (Figure 3C). Similar results were obtained in Hela-mtKeima cells, indicating enhanced mitophagy (Figure 3D). Next, we analyzed mitophagy level in HDFs by measuring the degradation of cytochrome C oxidase subunit II (COX II), a mtDNA-encoded inner membrane protein, following replicative senescence. Figure 3E shows that *Ap* or *Aa* treatment led to COX II degradation in sHDF. These findings suggest that *Ap* and *Aa* can activate mitophagy in various cell types, highlighting their potential for broader biological applications.

**Figure 4:**
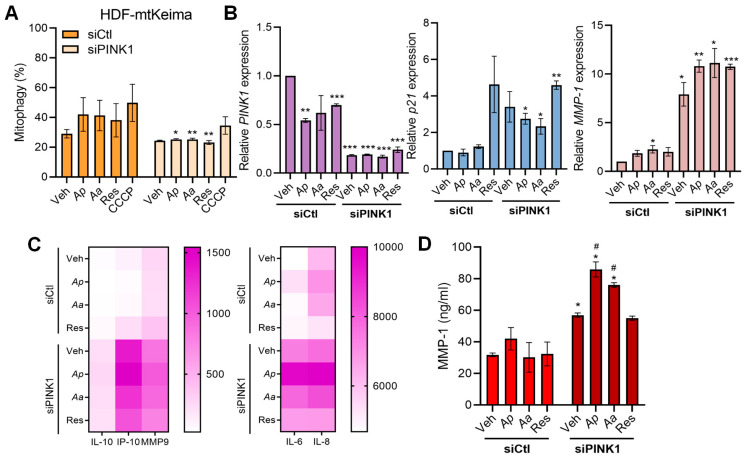
PINK1-mediated mitophagy regulates senescence phenotypes. Cells were transfected with control siRNA (siCtl) or PINK1 siRNA (siPINK1) and treated with vehicle (Veh), *Ap*, *Aa*, or resveratrol (Res) (10 μg/mL) for 24 h. (**A**) To quantify the level of mitophagy induction, HDF-mtKeima cells were quantified by flow cytometry based on mtKeima fluorescence ratio. CCCP (20 μM, 2 h) was used as a positive control for mitophagy induction. (**B**) PINK1 knockdown efficiency and mRNA expression levels of senescence markers *p21* and *MMP-1* were determined by qRT-PCR after 24 h of treatment. (**C**) Representative heatmap showing the expression level of SASPs-related cytokines analyzed by multiplex cytokine assay (Luminex). (**D**) MMP-1 protein secretion in the supernatant was quantified by ELISA. Statistical analysis: * *p* < 0.05, ** *p* < 0.01, *** *p* < 0.001 vs. siCtl-transfected vehicle-treated cells. # *p* < 0.05 vs. siPINK1-transfected vehicle-treated cells.

Since PINK1 is a key upstream regulator of canonical mitophagy initiation, we next sought to determine whether the mitophagy-activating and anti-senescence effect of starfish-derived extracts were dependent on PINK1 signaling. We first aimed to quantify the activation of mitophagy induced by *Ap* and *Aa* in the PINK1 knockdown condition using the HDF-mtKeima cell system. As a result, compared to the siCtl group, PINK1 knockdown significantly reduced the mitophagy-activating effects of the starfish extracts. These findings indicate that PINK1 is a key factor in *Ap*- and *Aa*-induced mitophagy activation, suggesting that *Ap* and *Aa* promote mitophagy in a PINK1-dependent manner (Figure 4A). Therefore, it was necessary to investigate the impact of PINK1 on the anti-senescence effects of *Ap* and *Aa*. To this end, we analyzed the expression of senescence-related genes in PINK1-knockdown HDFs following *Ap* or *Aa* treatment using qRT-PCR. First, to verify the efficiency of PINK1 siRNA-mediated knockdown, we assessed PINK1 mRNA expression and confirmed that its expression was effectively suppressed. Next, we examined the mRNA expression of the skin aging markers *p21* and *MMP-1* and found that their levels were elevated by at least 2-fold and up to 5-fold in the siPINK1 group compared to the siCtl group (Figure 4B). We next investigated whether the absence of PINK1 also affects SASP profiles. Under the same experimental conditions, we performed Luminex assays and ELISA using the supernatants collected from these samples. Through the results of the Luminex and ELISA assays, we found that PINK1 knockdown abolished *Ap*- or *Aa*-mediated regulation of pro-inflammatory cytokines, leading instead to an increase in their secretion. Moreover, MMP-1 secretion was significantly elevated under PINK1-deficient conditions. Taken together, these findings demonstrate that anti-inflammatory effects of the starfish extracts are mediated through the mitophagy key regulator PINK1 (Figure 4C,D). In summary, we demonstrate that PINK1, a key regulator of mitophagy, plays a critical role in the anti-senescence effects of the starfish extracts.

### 2.4. Starfish-Derived Extracts Improve Mitochondrial Function in Human Dermal Fibroblasts

**Figure 5:**
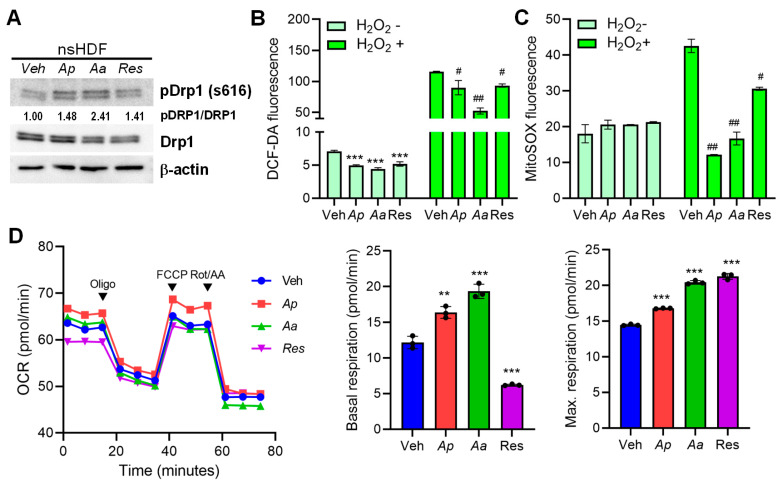
Starfish-derived extracts alters mitochondrial dynamics and improves mitochondrial functions. (**A**) Cells were treated with vehicle (Veh), 10 μg/mL of *Ap*, *Aa*, resveratrol (Res), or CCCP for 2 h. Protein levels of Dynamin-related protein 1 (Drp1) were analyzed by immunoblotting. Anti-β-actin antibody was used as a loading control. (**B**,**C**) *Ap*- or *Aa*-derived extracts treatment in HDF cells reduces reactive oxygen species (ROS) production in response to hydrogen peroxide (H_2_O_2_). To determine the intracellular antioxidant effect of *Ap*- or *Aa*-derived extracts, a 2′,7′-dichlorohydrofluorescein diacetate (DCF-DA) or mitoSOX fluorescent dye-containing media was added. Fluorescent intensity was measured and represented in the graph. (**D**) OCR were assessed over time, before or after addition of 2 μM oligomycin, 1 μM FCCP, 1 μM antimycin A and 1 μM rotenone and different metabolic parameters were assessed. All data were indicated as mean ± SD of at least three independent experiments. Statistical analysis: ** *p* < 0.01, *** *p* < 0.001 vs. Veh-treated cells. # *p* < 0.05, ## *p* < 0.01 vs. H_2_O_2_ treated cells.

Next, we wanted to evaluate the effect of starfish-derived extracts on the mitochondrial metabolism. Dynamin-related protein 1 (Drp1) is a central player in mitochondrial quality control, particularly through its role in mitochondrial fission. Drp1 is recruited from the cytosol to the outer mitochondrial membrane, where it assembles into spiral structures that constrict and divide mitochondria [26]. There was an increase in phosphorylation of Drp1, which is a key regulatory event that promotes mitochondrial fission, upon the treatment of starfish-derived extracts in HDFs (Figure 5A). Next we evaluated the intracellular antioxidant activity of starfish-treated HDFs in response to hydrogen peroxide (H_2_O_2_). Starfish-derived extracts treatment of cells alleviated the production of reactive oxygen species (ROS) and mitochondrial ROS production (Figure 5B,C). To determine whether Ap- or Aa-derived extracts lead to improved mitochondrial function, we measured their effects using Seahorse assay, particularly through the oxygen consumption rate (OCR), a key indicator of mitochondrial respiration. Starfish extracts increased basal respiration, as well as maximal respiratory capacity in sHDF (Figure 5D). Starfish-derived extracts’ ability to modulate mitochondrial respiration, as shown by oxygen consumption rate, supports its potential as a therapeutic agent in oxidative stress-related diseases.

## 3. Discussion

In this study, we demonstrate that extracts derived from *Asterias pectinifera* (*Ap*) and *Asterias amurensis* (*Aa*), two starfish species traditionally regarded as ecological threats, possess potent anti-aging properties in HDFs. Here, we report the ability of *Ap*- and *Aa*-derived extracts to promote mitophagy and reduce inflammation, suggesting a novel application for these marine organisms into valuable biosources for skin health. Our findings further highlight the multifaceted role of starfish-derived extracts in regulating mitochondrial homeostasis and cellular aging.

Mitophagy, the selective autophagic degradation of damaged mitochondria, plays a crucial role in maintaining mitochondrial quality and cellular homeostasis [27,28]. During senescence, mitophagy is often impaired, resulting in the accumulation of dysfunctional mitochondria that exacerbate oxidative stress and reinforce the senescent phenotype [17,18,29]. Our data indicate that sHDF treated with *Ap* and *Aa*-derived extracts exhibited a marked reduction in senescence-associated phenotypes, including decreased SA-β-gal activity, MMP-1 expression, and pro-inflammatory cytokines such as IL-6 and IL-8. These results suggest that the extracts exert anti-inflammatory effects, which are critical in mitigating skin aging. Considering that skin aging is a multifactorial process governed by complex interactions at both the epidermal and dermal levels, it will be important to investigate the effects of starfish-derived extracts on key epidermal functions, including keratinocyte proliferation, barrier integrity, and cellular differentiation. Such studies may reveal novel mechanisms by which these marine bioactive materials modulate skin physiology and contribute to anti-aging interventions.

Restoring mitophagy can attenuate senescence-associated features. Recent studies have highlighted that primary human cells maintain highly active basal mitophagy initiated by mitochondrial superoxide signaling and reactivation of mitophagy by a p62-targeting small molecule rescued markers of cellular aging [30]. Indeed, in UVB-irradiated HDFs, damaged mitochondria are removed by NIX/BNIP3L-dependent mitophagy [29]. This study aimed to elucidate whether starfish-derived extracts, beyond its reported anti-aging efficacy, modulates mitophagy in HDFs to alleviate senescence-associated inflammatory phenotypes and the expression of key senescence markers, including MMP-1 and p21. This suggests that mitophagy not only serves as a quality control mechanism but also actively modulates the molecular landscape of senescent cells. Beyond mitophagy, we observed that starfish-derived extracts enhance mitochondrial quality control through upregulation of phosphorylated Drp1, a key mediator of mitochondrial fission. This supports the notion that *Ap* and *Aa* promote mitochondrial dynamics, which are essential for maintaining mitochondrial integrity and function. Moreover, Seahorse assay revealed that *Ap* and *Aa* improve mitochondrial respiration, as indicated by increased basal and maximal oxygen consumption rates. This enhancement of mitochondrial bioenergetics suggests that these extracts not only remove damaged mitochondria but also support the functional capacity of the remaining mitochondria.

Several marine-derived compounds have been identified as modulators of mitochondrial function or signaling pathways, offering therapeutic potential against a range of human diseases, including neurodegenerative disorders, metabolic syndromes, and various forms of cancer [31,32,33]. Recently, marine-derived compounds have emerged as underexplored sources of bioactive molecules capable of modulating mitophagy pathways. One of the recent examples is PDE701, a diphenyl ether derivative isolated from the marine sponge *Dysidea* species. PDE701 selectively induces mitophagy and has a potential role in a chemotherapy-induced peripheral neuropathy model [34]. We previously published that collagen peptides extracted from *Asterias pectinifera* and *Halcynthia roretzi* have considerable potential for application in cosmetic formulations aimed at providing comprehensive skin protection, including antioxidant, anti-inflammatory, anti-photoaging, and antimicrobial benefits [6]. Another promising source of marine collagen is jellyfish, particularly species within the *Scyphozoa* class. Jellyfish are gaining increasing attention for their bioactive compounds with potential applications in biotechnology. Notably, Riccio et al. reported that specific jellyfish-derived fractions exhibit inhibitory effects on the proliferation of various human cancer cell lines, highlighting their therapeutic relevance [35,36]. Given these findings, it would be worthwhile to further explore whether other marine organisms might serve as novel sources of mitophagy-inducing agents, potentially opening new avenues for drug discovery and development.

Of note, we performed liquid chromatography–mass spectrometry (LC-MS) analysis of *Ap* and *Aa* extracts to provide insight into the rough composition of the extracts and confirm similar composition between *Ap* and *Aa* extracts (Supplementary Materials). Among starfish-derived components which could be responsible for mitophagy activation, we found a high level presence of choline, a metabolite known to be involved in modulating mitochondrial function. Given choline’s role as a substrate for choline dehydrogenase (CHDH), a key enzyme implicated in Parkin-mediated mitophagy [37], its presence in these starfish species suggests a potential link between choline metabolism and mitophagy activation. This finding raises the possibility that bioactive compounds derived from *Ap* and *Aa* may modulate mitophagy through CHDH-dependent pathways, offering a novel perspective on the molecular mechanisms underlying mitochondrial quality control by marine-derived drugs. Future investigation is warranted to confirm this hypothesis, specifically by assessing CHDH activity and the effects of purified choline within this model system.

In conclusion, starfish-derived compounds activate canonical mitophagy pathways, enhance mitochondrial quality control, and promote cellular antioxidant defenses, thereby mitigating senescence-associated phenotypes. By modulating PINK1-dependent mitophagy and promoting mitochondrial function, *Ap* and *Aa* offer a novel strategy for combating senescence and its associated pathologies. Future studies should explore their effects in in vivo models of skin aging, as well as investigate their potential synergy with other mitophagy modulators.

## 4. Materials and Methods

### 4.1. Preparation of Ap or Aa-Derived Extracts

*Asterias pectinifera* (*Ap*) and *Asterias amurensis* (*Aa*) were collected from the coastal waters of Dangjin, Republic of Korea. *Ap* and *Aa* were washed thoroughly, frozen, and stored at −20 °C until further use. Each species was immersed in a 7% aqueous potassium hydroxide (KOH) solution at a 1:1 ratio (*w/v*) for 24 h at room temperature to remove non-collagen substances. The resulting materials were rinsed with distilled water until the pH reached 8.0. *Ap* and *Aa* ossicle extract was mixed with distilled water at a ratio of 1:10 (*w/v*). DL-tartaric acid and ascorbic acid were added to the mixture at final concentrations of 0.65% (*w/v*) each. The sample was subjected to ultrasonic treatment at 20% Ampl for 30 min using an ultrasonic processor (HD4400, Bandelin, Berlin, Germany). The solution was stirred at 4 °C, and its pH was adjusted to 7.0 during stirring. Subsequently, complex protease ZF101, (Angel Yeast Co., Ltd., Yichang, Hubei, China) was added at a concentration of 0.01% (*w/v*), and enzymatic hydrolysis was carried out by continuous stirring at 4 °C for 24 h. After removing the precipitation by centrifugation, the mixture was heated at 80 °C for 5 min to inactivate the enzyme. The resulting starfish-derived extracts were lyophilized using a freeze dryer (TFD8501, Ilsinbiobase, Dongducheon, Korea) for 72 h. The lyophilized powders were reconstituted in distilled water filtered through a 0.22 µm pore-size filter unit (Corning Inc., Corning, NY, USA, Cat. No. 431097) and used as samples in subsequent experiments.

To conduct a qualitative analysis of starfish extracts, samples were analyzed using a Vanquish UHPLC system (Thermo Fisher Scientific, Waltham, MA, USA) coupled with a heated electrospray ionization (H-ESI) source. Briefly, starfish extracts were centrifuged at 12,000× *g* for 10 min at 4 °C and filtered through a 0.22 µm PTFE syringe filter prior to analysis. Chromatographic separation was achieved on a Hypersil Gold C18 column (100 × 2.1 mm, 1.9 µm) maintained at 40 °C. The mobile phases consisted of solvent A (0.1% formic acid in water) and solvent B (0.1% formic acid in methanol), delivered at a flow rate of 0.3 mL/min under the following gradient: 0–3 min, 90% A; 3–13 min, 10–100% B; 13–17 min, 100% B; 17–17.5 min, 100–10% B; 17.5–20 min, 10% B. The injection volume was 5 µL. Mass spectrometric detection was performed with an Orbitrap analyzer at a resolution of 120,000 over an m/z range of 70–1000. Ionization was carried out in both positive (3.5 kV) and negative (2.5 kV) modes. Data-dependent MS/MS fragmentation was conducted using a normalized collision energy (NCE) of 30. Compound identification was performed by comparing accurate mass and MS/MS spectra with reference databases using Thermo Scientific Compound Discoverer 3.3 software.

### 4.2. Replicative Senescence Cell Model

HDF cells were cultured in RPMI-1640 (Corning Inc., Corning, NY, USA) supplemented with 10% fetal bovine serum (FBS) (GenDEPOT, Barker, TX, USA), 25 mM HEPES (GenDEPOT, Barker, TX, USA), 100 U/mL penicillin, and 100 µg/mL streptomycin (Gibco, Grand Island, NY, USA). Cells were maintained at 37 °C in a 5% CO_2_ incubator and passaged every 3–4 days. A replicative senescence model has been introduced previously. HDF cells were cultured beyond passage 20 to induce replicative senescence, following a method similar to that previously described [38]. Senescence was quantified by SA-β-galactosidase staining kit (Cell Signaling Technology, Danvers, MA, USA; 9860) following the manufacturer’s instructions.

### 4.3. Cell Viability Assay

HDF cells were seeded in 96-well plates. After 24 h, the medium was replaced, with or without *Ap* or *Aa* in various concentrations. To measure cell viability, at 72 h after treatment, the old medium was removed and cells were incubated with CCK8 (CK04-11, Dojindo, Kumamoto, Japan) solution for 1 h. The absorbance at 450 nm was measured using a Varioskan™ LUX multimode microplate reader (Thermo Scientific, Waltham, MA, USA).

### 4.4. Quantification of Mitophagy Activity Using mtKeima

To quantify the induction level of mitophagy, HDF and Hela stably expressing mt-Keima were generated (a kind gift of Prof. Jinho Yun, DongA University School of Medicine, Korea) and analyzed using an LSR Fortessa X-20 flow cytometer (BD Biosciences, San Jose, CA, USA) equipped with a 405 nm/561 nm laser and BV605/PE-CF594 detector, as described previously [34,39]. The percentage of cells undergoing mitophagy was determined by gating cells exhibiting a high ratio of emission at 561 nm/405 nm excitation.

### 4.5. ROS Measurement Assay

To measure intracellular ROS, cells were seeded in a 96-well plate. After Veh, *Ap*, *Aa*, or resveratrol treatment with or without H_2_O_2_, cells were stained with 10 μM 2′,7′-dichlorofluorescein diacetate (DCF-DA; Sigma-Aldrich, St. Louis, MO, USA) in serum-free (SF) media. For mitochondrial-mediated ROS measurement, 1 μM MitoSOX™ Green (Invitrogen, Waltham, MA, USA) in SF media was incubated with cells for staining. After 30 min, the medium was removed, and the cells were washed with phosphate-buffered saline (PBS). Fluorescence was measured using a Varioskan™ LUX multimode microplate reader (Thermo Scientific) at 490 nm excitation/520 nm emission for DCF-DA, and 488 nm excitation/510 nm emission for MitoSOX Green. For additional analysis of mitochondrial ROS, MitoSOX Green-stained cells were also analyzed by flow cytometry using an LSR Fortessa X-20 equipped with a 488 nm laser and FITC detector.

### 4.6. NF-κB Luciferase Reporter Assay

HDF cells were transfected with pNF-κB-Luc plasmid using Lipofectamine 2000 (Thermo Fisher Scientific) according to the manufacturer’s protocol. After 24 h, cells were treated with vehicle, *Ap* (10 μg/mL), *Aa* (10 μg/mL), or resveratrol (10 μg/mL), either in the presence or absence of lipopolysaccharide (LPS; 100 ng/mL) co-treatment to stimulate NF-κB activity. NF-κB transcriptional activity was measured 24 h after treatment using a Dual-Glo luciferase assay kit (Promega, Madison, WI, USA).

### 4.7. Quantitative Real-Time Polymerase Chain Reaction (qRT-PCR)

Total RNA was isolated using a Direct-zol RNA Miniprep Kit (Zymo Research, Irvine, CA, USA; R2050) following the manufacturer’s instructions. RNA quality was assessed using a NanoDrop ND-2000c (Thermo Fisher Scientific). cDNA synthesis was performed using the High-Capacity cDNA Reverse Transcription Kit (Applied Biosystems, Waltham, MA, USA; 4368814), and relative mRNA levels were analyzed on the QuantStudio 1 Real-Time PCR System (Applied Biosystems) with the Power SYBR Green PCR Master Mix (Applied Biosystems; 4368706). The GADPH gene served as housekeeping control. Primer sequences were previously reported [40].

### 4.8. Measurement of Cytokines and Chemokine Secretion

IL-6, IL-8 and MMP-1 were measured from cell supernatants using R&D ELISA kits (R&D Systems, Minneapolis, MN, USA). ELISA was performed using the manufacturer’s instructions, and absorbance at 450 nm was measured using a microplate spectrophotometer. For multiple screening, a magnetic Luminex screening assay with a Human Premixed Multi-Analyte Kit (R&D Systems) was used.

### 4.9. siRNA Transfection

HDF cells were transfected with control siRNA or PINK1-specific siRNA (Bioneer, Daejeon, Korea) using Lipofectamine RNAiMAX (13778100, Invitrogen) according to the manufacturer’s instructions.

### 4.10. Immunoblot Analysis

Protein samples were separated by 8–15% SDS-PAGE and transferred to polyvinylidene difluoride membranes (PVDF; IPVH00010, Millipore, Burlington, MA, USA). Membranes were blocked for 1 h at room temperature in 5% skim milk prepared in Tris-buffered saline containing 0.1% Tween-20 (TBS/Tw; T2008, Biosesang, Youngin, Korea) and then incubated RT 1 h or overnight at 4 °C with primary antibodies. The following antibodies were used: phospho-p44/42 MAPK (Erk1/2) (Thr202/Tyr204) (9101, Cell Signaling Technology), p44/42 MAPK (Erk1/2) (137F5) (4695, Cell Signaling Technology), phospho-DRP1 (Ser616) (3455, Cell Signaling Technology), DRP1 (D8H5) (5391, Cell Signaling Technology), COX II (ab198286, Abcam, Cambridge, UK), and β-actin (AM1021B, Abcepta, San Diego, CA, USA). After washing, membranes were incubated with HRP-conjugated secondary antibodies (anti-mouse or anti-rabbit) and visualized using the Fusion Solo Imaging System (Vilber Lourmat STE, Collegien, France). Band intensities were quantified using ImageJ software (version 1.53e) (National Institutes of Health, Bethesda, MD, USA).

### 4.11. Confocal Microscopy

HDF cells were treated with vehicle, *Ap*, *Aa*, or resveratrol for 24 h, then incubated in serum-free medium containing MitoTracker Green (Thermo Fisher Scientific) and LysoTracker Deep Red for 30 min. Mitochondrial and lysosomal signals were subsequently visualized using a confocal microscope (LSM900, Carl Zeiss, Oberkochen, Germany).

### 4.12. Transmission Electron Microscopy (TEM)

HDF cells were treated with vehicle, *Ap*, *Aa*, or resveratrol. Cells were then fixed in 2.5% glutaraldehyde for 12 h, followed by post-fixation in reduced osmium tetroxide for 1.5 h at 4 °C. After three washes, samples were dehydrated through a graded ethanol series and embedded in Epon resin. Ultrathin sections were prepared, stained with uranyl acetate and lead nitrate, and examined using a Hitachi H-7000 transmission electron microscope(Hitachi High-Tech Corporation, Tokyo, Japan) to visualize autophagosome formation and assess mitochondrial ultrastructure.

### 4.13. Mitochondrial Respiration Analysis

Cells were plated in mini-plates (Agilent, Santa Clara, CA, USA, Cat. No. 103022-100) at 10,000 cells/well and placed in a non-CO_2_ incubator for 1 h prior to oxygen consumption rate (OCR) measurement. The Seahorse XFp Cell Mito Stress Test Kit (Agilent, Cat. No. 103010-100) was employed following the manufacturer’s protocol. In brief, basal OCR was recorded first, followed by sequential injections of 1 μM oligomycin, 2 μM fluoro-carbonyl cyanide phenylhydrazone (FCCP), and a mixture of 1 μM rotenone with 1 μM antimycin A at the designated assay intervals.

### 4.14. Statistical Analysis

All data are expressed as the mean ± standard deviation (SD) from a minimum of three independent experiments. Statistical significance between treatment groups was assessed using either the Mann–Whitney U test, two-tailed unpaired Student’s *t*-test, or two-way ANOVA, as appropriate. Significance levels are indicated as follows: * *p* < 0.05; ** *p* < 0.01; *** *p* < 0.001; ns, not significant. Graph generation and statistical analyses were conducted using GraphPad Prism 9 software (GraphPad Software, San Diego, CA, USA).

## Data Availability

The data presented in this study are available on request from the corresponding author.

## Supplementary Materials

The following supporting information can be downloaded at https://www.mdpi.com/article/10.3390/md23110418/s1. Table S1: List of compounds identified by LC-MS.
